# Infected closed pelvic fractures in two adults - Case report

**DOI:** 10.1016/j.ijscr.2024.110142

**Published:** 2024-08-09

**Authors:** Jimmy Olomi, Joseph Msemwa, Baraka Mponda

**Affiliations:** University of Dar es Salaam, Tanzania; Mbeya Zonal Referral Hospital, Tanzania

**Keywords:** Closed fractures, Infected fractures, Infected closed fractures, Infected pelvic fracture, Case report

## Abstract

**Introduction:**

Infections following closed fractures in immunocompetent adults are rare but can have significant consequences if not promptly diagnosed.

**Case presentation:**

We present two cases of immunocompetent adults admitted to a hospital in Tanzania with closed pelvic fractures who were found to have infections intraoperatively. Both patients responded well to treatment with one delaying to clear the infection.

**Clinical discussion:**

Because it rarely happens infected closed fractures are difficult to diagnose prior to surgery and treatment is controversial, most authors propose priority should be getting the fracture to unite then treatment of infection.

**Conclusion:**

This report adds to the existing literature on infections following closed fractures and highlights the importance of considering infection in closed fractures and tailoring management strategies to individual patient factors.

## Highlights

• Infection in closed fractures a rare occurance.
• The use of anti TB therapy in the management of bone infection
• Fracture stability is important while dealing with infection.

## 1 Introduction

Infection following a closed fracture is an uncommon occurrence, primarily documented in pediatric populations ([1], [2], [3]) and immunocompromised individuals (4,5). While in open fractures the pathogenesis of infection is well established, in closed fractures it remains less understood.

In pediatric cases a hematogenous spread from distant sites, including the respiratory and urinary tracts, is suspected to be the source of infection. Immunocompromised individuals, such as those with diabetes, HIV, or using immunosuppressive medications such as steroids, and chemotherapy are particularly vulnerable to infections as their immunity is low and their ability to resist infection is compromised (6).

Reported instances in immunocompetent adults are rare, with only a few cases involving bones such as the humerus, distal radius, tibial plateau, patella and femur ([6], [7], [8], [9]) and none in Africa.

Here, we present two cases of immunocompetent adult patients admitted with closed pelvic fractures who were found to have infections intraoperatively, this raises awareness to the possibility of infection in closed fractures and adds to the existing body of knowledge on the condition, its existence and possible treatment options. we present these cases according to the updated SCARE 2023 Guidelines (10).

## 2 Case presentation

### 2.1 Case 1

**Fig. 1:**
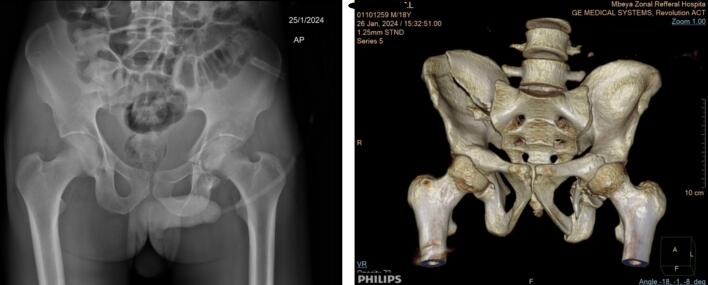
Left; A plain radiograph of the patient showing a right iliac wing fracture, which is not clearly visible due to bowel overshadowing and a right acetabular anterior and posterior column fracture. Right; A 3D reconstruction image revealing a comminuted displaced fracture of the right iliac bone extending to the sacroiliac joint (SIJ) articular surface, along with left acetabular anterior and posterior column fractures and a quadrilateral plate fracture.

A 24-year-old male from Ethiopia was involved in a motor vehicle accident as a passenger and sustained injury to his pelvis, resulting in severe pain and inability to bear weight, without other associated injuries. On admission, he was afebrile with a temperature of 36.5 °C and had stable vital signs. Pelvic X-rays and a CT scan revealed a comminuted right iliac wing fracture and left acetabular fracture (Fig. 1).

Laboratory tests showed a slightly elevated white blood cell (WBC) count of 11.6 × 10^9/l, with a predominant neutrophil count of 75.8 % (8.46 × 10^9/l). His hemoglobin level was 12.0 g/dl, and the platelet count was 127 × 10^9/l.

**Fig. 2:**
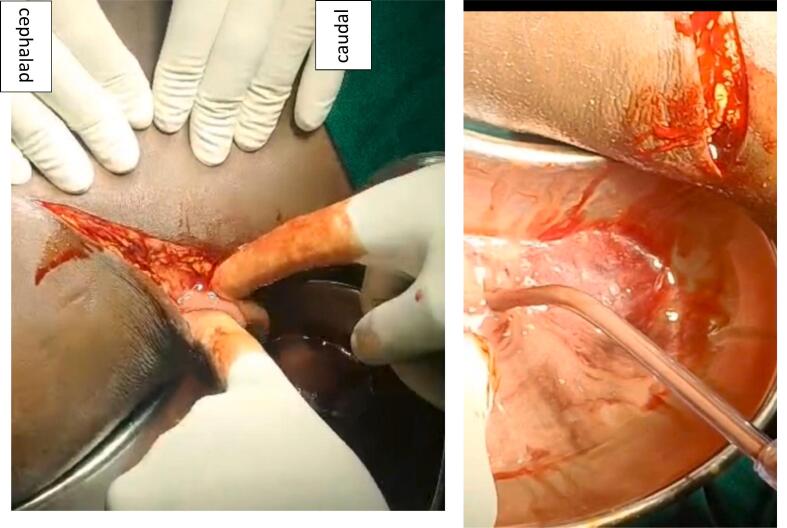
Intraoperative images showing pus drainage from the incision after incising the fascia (left) and collected pus in a kidney dish (right).

Two days after admission the patient's pain was increasing despite administration IM Diclofenac 75 mg 8hrly with IV Pethidine 100 mg 8hrly. The patient was taken to the operating room for pelvic reconstruction with plates and screws. Intraoperatively, the patient in supine position, an anterior approach to the iliac wing and sacroiliac joint was used. Upon opening the fascia overlying the external oblique muscle, approximately 550 ml of pus was drained under pressure (Fig. 2). A swab for culture was taken, debridement and irrigation with copious amount of saline was done and the fracture was stabilized with two 3.5 mm cortical screws. Postoperatively, the patient was kept on intravenous (IV) Chloramphenicol 1 g 12hrly and IV Metronidazole 500 mg 8hrly empirically and underwent regular wound care and monitoring for signs of infection his body temperature ranged from 36.8^0^C to 37.6^0^C. Day 7 post operation culture grew staphylococcus aureus which was sensitive to chloramphenicol.

This patient's postoperative recovery was uneventful. At the time of discharge, 7 days post-surgery, the wound was dry with no local signs of infection, and had begun ambulating with crutches. The patient was prescribed oral Chloramphenicol 250 mg every 8 h for 6 weeks. At the 14-day outpatient follow-up, the wound remained dry with no signs of infection, and the patient was ambulating with minimal pain. Unfortunately, further follow-up was not possible as the patient returned to his home country, Ethiopia.

### 2.2 Case 2

**Fig. 3:**
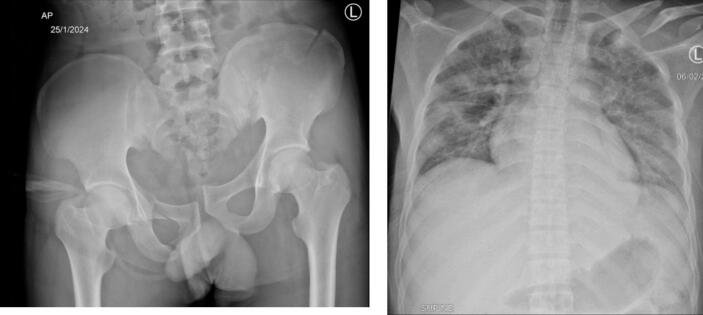
An anteroposterior (AP) pelvic radiograph, revealing a pelvic ring fracture(left) and a posteroanterior (PA) chest radiograph (right) shows a left clavicle fracture.

A 23-year-old Ethiopian male, a fellow passenger, sustained injuries to his pelvis and left clavicle (Fig. 3), with no other associated injuries. Upon admission, he was afebrile with a temperature ranging from 36.8 °C to 37.2 °C, but had a raised pulse of 100–106 beats per minute. Despite receiving analgesics (IM Diclofenac 75 mg 8hrly and IV Pethidine 100 mg 8hrly), he complained of severe pain.

After assessment, it was decided to treat his pelvic fracture operatively and the clavicle non operatively.

**Fig. 4:**
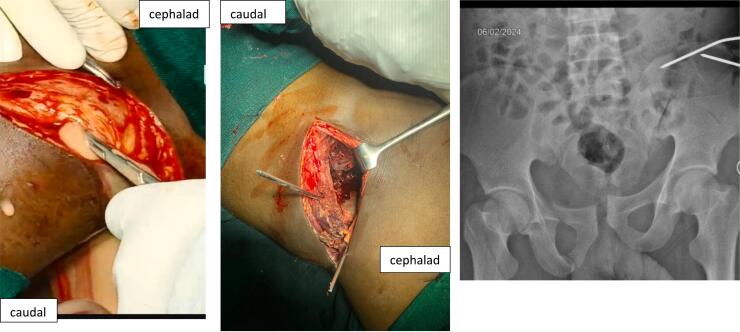
Left: Image captured after making the incision and opening the fascia over the external oblique muscle. Middle: Image taken after surgical debridement and k-wire fixation. Right: AP pelvic radiograph following temporary k-wire fixation.

**Fig. 5:**
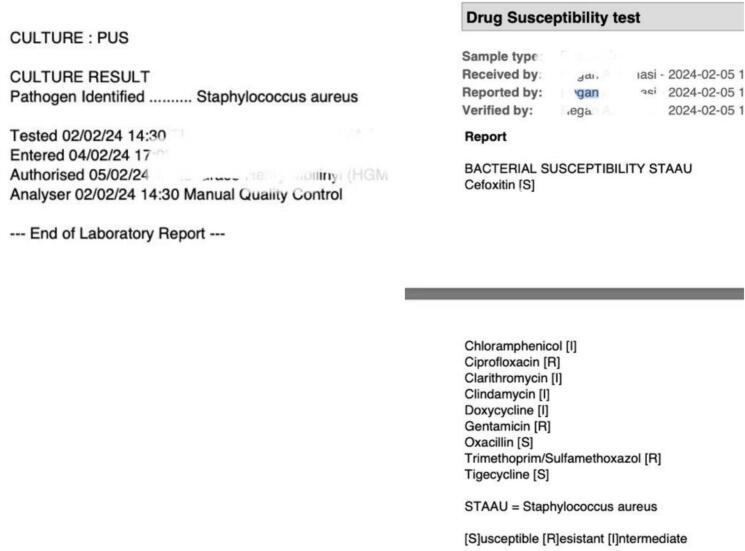
displays the results of the pus culture and sensitivity testing. The culture revealed the presence of *Staphylococcus aureus*, and the sensitivity results indicated the antibiotic susceptibilities of the isolated strain.

**Fig. 6:**
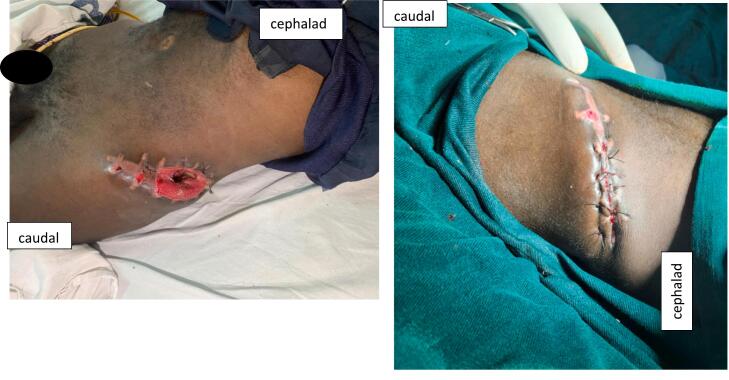
picture of the surgical site 5 weeks post debridement (Left) and picture of the wound after secondary suturing (Right).

Two days after admission, he was taken to the operating room and approximately 350 mls of thick pus under pressure was drained upon opening the fascia (Fig. 4). Debridement and irrigation with 6 l of saline were done. Instead of using plates and screws for fixation, the fractures were temporarily stabilized with two 3 mm K wires (Fig. 4). The patient was started on empirical IV Chloramphenicol 1 g 12hrly and IV Metronidazole 500 mg 8hrly. Culture results later revealed the growth of *Staphylococcus aureus* resistant to most available antibiotics (Fig. 5). The patient continued to experience pain and had fevers with a temperature of 38.8 °C. A hyperemic and tender swelling on the clavicle was also noted, which after incision pus was drained. Subsequently, oral fixed-dose combination of Rifampicin, Isoniazid, Ethambutol, and Pyrazinamide (RHZE), used for tuberculosis treatment in Tanzania, was initiated. The patient responded well to this treatment, with no further discharge from his wounds after a week. After a total of 5 weeks, he underwent removal of the k wires and secondary suture (Fig. 6). Further fixation was not performed as the fracture had stayed for more than 4 weeks and had begun ambulation with minimal pain. He was discharged after 6 weeks his wounds had healed well with no discharge or other signs of infection at this point, he was also taken back to Ethiopia.

## 3 Discussion

Infections following closed fractures in immunocompetent adults are rare, as evidenced by the limited availability of data and to the best of our knowledge only case reports available. This rarity, coupled with the overlap of inflammatory markers as in trauma and fractures, with those of infection, infection is less suspected in patients with closed fractures. However, a high index of suspicion is crucial, as it is now clear infection should never be ruled out in these cases.

From reported cases, it is evident that a typical picture of infection may not be present ([6], [7], [8]). For example, in the cases infected closed patella and tibial plateau fractures, authors report, patients did not exhibit fever or other physical signs of infection, and the diagnosis was only made during surgery (6,7). In our cases, initial presentation showed no typical signs of infection either, however, on retrospective analysis, infection may be attributed to the unresolving pain and tachycardia that the patients were presenting with.

In the reported case of a closed humerus fracture the patient presented with one episode of fever spike which was not enough to suspect infection (8).

Infection poses challenges in fracture management, especially when diagnosed prior to fixation, as there is no guideline for this, however, common approach is to use external fixation until the infection clears, followed by definitive fixation as in open fractures with severe contamination (11), However, an expert review on infection after fixation of fractures (IAFF) revealed that most surgeons prefer to do debridement and retain implants until the fracture heals. The review emphasizes that while eradicating infection and biofilm is important, the primary goal should be to achieve fracture healing (12).

In recent reported cases of infection following closed fractures, surgeons opted for definitive fixation without using temporizing measures. All patients in these cases healed and cleared the infection but they all had to undergo another round of debridement with retention of the implants, except for one with a humerus fracture who during the revision surgery exchange of plates was done after debridement ([6], [7], [8]). In our cases, both approaches were used. In the first case, definitive stabilization with screws was successful, and the patient had a good short-term outcome. However, in the second case, where temporary K-wires were used, the patient had a delayed control of infection and this led to the prolonged period of waiting while the patient is on k wires.

In the setting of infection, antibiotics must be provided alongside stable fixation of the fracture, and a proper choice of antibiotics is crucial for infection eradication. Performing a culture as soon as infection is diagnosed is important.

In the second case, when the patient was switched to anti-tuberculosis drugs (usually reserved for tuberculosis treatment in Tanzania), there was a very dramatic response. Rifampicin has been used for the treatment of prosthetic joint and bone infections and has been recommended in several guidelines, including the American and French guidelines (13,14). In a retrospective study Tonnelier M. et al. showed that rifampicin, even at low doses, may be effective in treating bone infections (15).

## 4 Conclusion

Infections following closed fractures in immunocompetent adults are rare and signs may not be typical, a high index of suspicion is essential, and a possibility of infection should never be ruled out in closed fractures.

The approach to managing these infections is not standardized, our experience underscores the importance of selecting the appropriate management strategy based on individual patient factors and the need for vigilant antibiotic therapy. And that in cases where infection persists, switching to alternative antibiotic regimens, such as anti-tuberculosis drugs, may yield favorable outcomes.

Author contribution

Authors

Jimmy Olomi

• Writing of the manuscript
• Operated on the patients
• patient followup

Joseph Msemwa

• Reviewed the manuscript
• lead surgeon in one of the patients

Baraka Mponda

• Reviewed the manuscript
• lead surgeon in one of the case
• Follow up on the patient

Contributors

• Benedicto Minga
• Rosemary P Kiwelu

## Guarantor

Jimmy Olomi

## Declaration of competing interest

The authors declare that they have no known competing financial interests or personal relationships that could have appeared to influence the work reported in this paper.
